# No Evidence for Contagious Yawning in Juvenile Ravens (*Corvus corax*): An Observational Study

**DOI:** 10.3390/ani12111357

**Published:** 2022-05-26

**Authors:** Andrew C. Gallup, Anja B. Schild, Markus A. Ühlein, Thomas Bugnyar, Jorg J. M. Massen

**Affiliations:** 1 Psychology and Evolutionary Behavioral Sciences Programs, SUNY Polytechnic Institute, Utica, NY 13502, USA; 2 Department of Biological Sciences, Nova Southeastern University, Ft. Lauderdale, FL 33314, USA; 3 Department of Behavioral & Cognitive Biology, University of Vienna, 1090 Vienna, Austria; aschild@zedat.fu-berlin.de (A.B.S.); markus.uehlein@lifebrain-labor.at (M.A.Ü.); thomas.bugnyar@univie.ac.at (T.B.); 4 Animal Behaviour and Cognition, Utrecht University, 3584 CS Utrecht, The Netherlands

**Keywords:** collective behavior, comparative cognition, motor synchrony, social behavior, state change

## Abstract

**Simple Summary:**

Research suggests that contagious yawning evolved to synchronize activity and vigilance within groups. To date, yawning has shown to be contagious in a wide range of mammalian species, including humans, great apes, some monkey species, domesticated dogs and pigs, wolves, lions, and rats. However, yawn contagion has only been previously documented in one bird species: budgerigars (*Melosittacus undulatus*). Here, we sought to examine whether yawning was contagious among juvenile common ravens (*Corvus corax*). By observing two small and undisturbed captive groups, we also assessed the contagious nature of other frequently observed and potentially contagious behaviors: stretching, scratching, and shaking. Overall, we found no evidence for behavioral contagion. Given the limitations to the observational methods, however, we suggest future experimental research be conducted to support these findings.

**Abstract:**

The overt and reflexive matching of behaviors among conspecifics has been observed in a growing number of social vertebrates, including avian species. In general, behavioral contagion—such as the spread of yawning—may serve important functions in group synchronization and vigilance behavior. Here, we performed an exploratory study to investigate yawn contagion among 10 captive juvenile ravens (*Corvus corax*), across two groups. Using observational methods, we also examined the contagiousness of three other distinct behaviors: stretching, scratching, and shaking. A total of 44 20 min observations were made across both groups, including 28 in the morning and 16 in the afternoon. The time and occurrence of all the behaviors from each bird were coded, and the temporal pattern of each behavior across both groups was then analyzed to assess the degree of social contagion. Overall, we found no evidence for contagious yawning, stretching, scratching, or shaking. However, yawns were relatively infrequent per observation (0.052 ± 0.076 yawns/bird) and thus experimental methods should be used to support this finding.

## 1. Introduction

The overt and reflexive matching of behaviors among conspecifics, also referred to as behavioral contagion [1], is common among social species and could provide fitness advantages to group members [2]. In particular, contagious behaviors may serve important functions in synchronizing activity patterns and facilitating collective vigilance within groups [3,4,5]. Although the study of contagious behaviors has focused primarily on mammalian species [6,7,8,9,10], a number of studies have also found evidence for behavioral contagion among birds [11,12,13,14,15,16].

Perhaps the exemplar of contagious behavior is yawning. In humans, seeing, hearing, or even thinking about others yawning triggers contagion [17,18,19]. Among non-human primates, experimental evidence for contagious yawning has been documented in chimpanzees [6,20,21], bonobos [22], and orangutans [23]. Experimental studies have also shown evidence for yawn contagion in a subline of high-yawning Sprague Dawley rats [24] as well in as domesticated dogs in response to human yawns [25], but not to conspecifics [26]. Yawn contagion has been further reported within observational studies of gelada baboons [27], captive wolves [28], domesticated pigs [29], and African lions [3]. Limited evidence for contagious yawning has also been documented in African elephants [30], southern elephant seals [31], and domesticated sheep [32]. Some other mammalian species that have been studied for yawn contagion, but have revealed no evidence for this effect, include common marmosets, ring-tailed and ruffed lemurs, as well as lowland gorillas [33,34,35].

To date, the only evidence for contagious yawning in a non-mammalian species is in budgerigars (*Melopsittacus undulatus*). An initial observational study found that yawns were temporally clustered in a captive flock, and that the clumping of yawns could not be explained by circadian factors [36]. A subsequent study confirmed the presence of yawn contagion in this species, using experimental manipulations which included both live interactions and video recordings of conspecifics [37]. To our knowledge, no other species of bird has been tested for contagious yawning; thus, it remains likely that yawn contagion is also prevalent within this taxon.

One avian species that has been of particular interest in behavioral research is common ravens (*Corvus corax*). Ravens are a moderately social species, with non-breeders regularly forming groups during foraging and roosting [38,39]. These birds remain keenly attuned to the behaviors of group members [40], and previous studies have shown that they display collective behaviors in flock formation [41] and feeding recruitment [42], can cooperate extensively [43], and are able to coordinate the necessary actions for cooperation [44]. Moreover, recent studies have shown that ravens synchronize their play behavior [45] and display contagious allopreening [46]. However, some of the more prototypical contagious behaviors, such as yawning, have yet to be examined in this species. Ravens represent a good candidate for the study of contagious yawning, given that this response has been linked with empathy and emotional contagion [7,8,47,48], and recent studies have shown that these birds display both positive and negative forms of emotional contagion [49,50]. Similarly, ravens show complex social cognition [38,40,51,52], and some of their socio-cognitive skills, such as consolation and emotional contagion [49,53], seem to be linked to empathy, to some degree [28,54].

Therefore, the current study sought to investigate the presence of contagious yawning in captive groups of juvenile common ravens. Raven juveniles spend the first years of their lives in flocks, before they might establish a pair bond and acquire a territory. The juvenile period is, in fact, the most social period for ravens [39], and in these flocks, they form multiple differential social bonds. Thus, during this developmental stage, these birds would specifically benefit from any advantages related to behavioral contagion, making the animals selected for this study the best sample for examining contagion.

In addition to yawning, the contagiousness of the following three behaviors was also examined: stretching, scratching, and shaking. Stretching is often associated with yawning across species [55,56] and, in birds, may function in promoting preparation for flight. In addition, similar to yawning, stretching has previously been shown to be contagious among budgerigars [36,57,58]. Scratching is another behavior known to be contagious both in humans [59] and other mammalian species [31,60,61] but, to date, has not been examined among birds. Similar to stretching, contagious scratching has previously been studied alongside yawn contagion [62]. In addition, mirror neurons have also been implicated in both responses [63,64]. Lastly, shaking behavior, representing a conspicuous shuttering of the feathers, was examined, due to its common occurrence and potential links to arousal and group activity [65].

Using observational methods from previous studies of behavioral contagion in budgerigars [36] and marmosets [33], we examined the temporal distribution of each of these four behaviors to test for the presence of non-random clustering or clumping through behavioral runs that would be indicative of contagion. In addition to assessing the social influence on these responses, the naturalistic frequency and circadian variation of these four behaviors was examined for the first time.

## 2. Materials and Methods

### 2.1. Subjects

**Figure 1:**
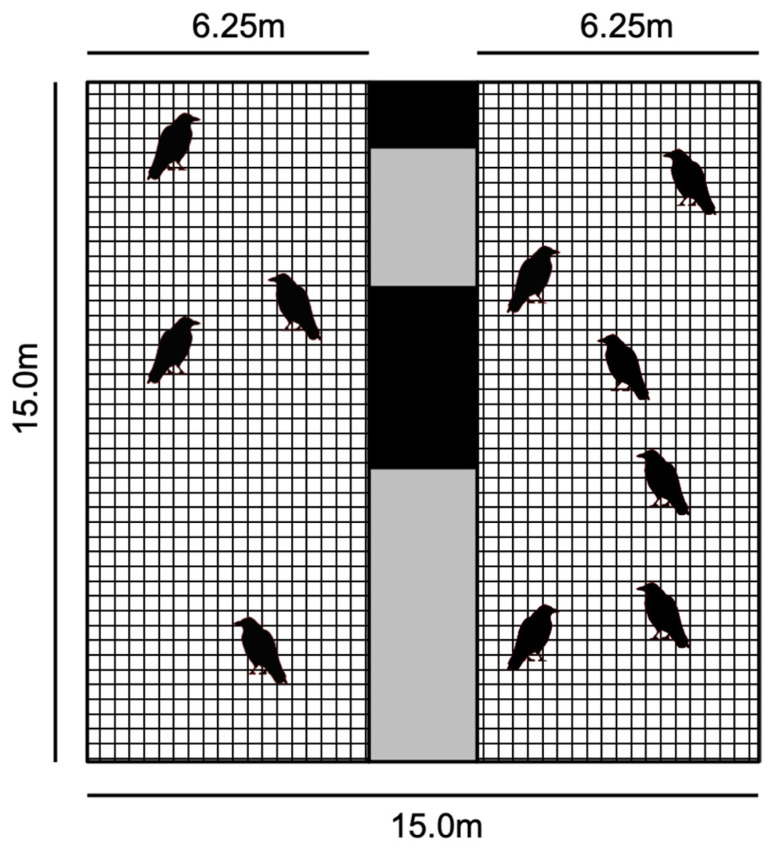
Schematic of the wire-mesh testing enclosures for both groups of birds. Black areas depict opaque barriers within the 2.5 m of separation between groups during testing.

A flock of ravens (7 males and 3 females), housed at Haidlhof research station (±35 km South of Vienna, Austria), were used for this study. All individuals were juvenile, non-breeding, approximately 2 years of age, and had been living in this flock for 2 years. The birds were hand-raised socially, i.e., together with peers, and thus, just like in nature, their development was social, which should have facilitated behavioral contagion, if present. The study was conducted between March 2014 and June 2014. The birds were housed in an outdoor aviary (Figure 1), fed twice a day on a well-balanced and mixed diet with a high meat content, and were provided water *ad libitum*.

On each testing day, the subjects were separated into 2 groups for better observation. We grouped the birds according to their social relationships to avoid stress during the observations, though this resulted in unequal numbers (Group 1 contained four birds; Group 2 contained six birds). The two groups remained consistent in all cases but one, where we were unable to separate one bird for testing. Once separated, the groups were kept split in two adjacent outdoor aviary compartments during all observation periods, and then reunited at the conclusion of testing each day. While there was some visual access between the two groups, this was mostly obscured. Approximately 1/3 of the barrier between the groups was opaque, and the remaining 2/3 consisted of two wire-mesh barriers with 2.5 m of separation (see Figure 1).

### 2.2. Procedure

We performed observations of the undisturbed groups to characterize any natural behavioral patterns of yawning, stretching, scratching, and shaking. The observations took place with two human observers seated in front of the aviary with a Canon HF-20 HD-Recorder (Canon U.S.A., Inc., Melville, NY, USA). A total of twelve testing sessions were performed, which included two distinct 20 min observations of each group, though one testing session had to be excluded due to heavy rain, which caused the ravens to hide in the back area of the aviary. From the remaining 11 testing sessions, 7 took place in the morning and 4 in the afternoon. Each testing session lasted 90 min and consisted of four separate behavioral observations, with each observation lasting 20 min. The first two observations from each testing session were successive; the first was conducted on Group 1 and the second was conducted on Group 2. A 10 min break then followed before two additional successive observations were performed, again, with the first on Group 1 and the second on Group 2. Thus, all observations were independent. Morning observations took place from 9:00 to 11:30 h and afternoon observations took place between 15:00 and 18:30 h. During observations, all the birds were video recorded, and the behavioral data were coded instantly on a sheet, since the enclosure size and the positioning of the seating branches did not always allow the camera a complete view of each individual.

### 2.3. Behavioral Coding

Video data were transferred to a computer and analyzed using video software (VLC Mediaplayer). A.B.S. and M.A.Ü., who both received training to recognize each behavior beforehand, coded each session together for the time and occurrence of all behaviors. Since the behaviors of interest were clearly defined and very straightforward to observe, we did not have any drift in the identification of behavior. No inter-rater reliability statistics were conducted, since all behaviors were rated in the presence of both observers, with consensus. A description of each of the behaviors is provided in Table 1.

### 2.4. Analysis

First, the overall frequency of each behavior was calculated per bird and then separately, across the morning and afternoon observations. To assess potential differences in these frequencies between the morning and afternoon testing sessions, as well as between males and females, we ran a Generalized Linear Mixed Model (GLMM) with a logit link function in R-studio [66], using the lme4 package [67]. This allowed us to account for the nested structure of our data (i.e., the two different groups) by adding ‘group’ as a random variable. The time of day and the sex were subsequently added as fixed factors.

The analyses of contagion followed similar methods to Miller et al. [36] (for budgerigars) and Massen et al. [33] (for marmosets). To rule out circadian factors as contributing to a temporal clumping or clustering of behaviors, first, the distribution frequencies for each behavior were plotted at 5 min intervals for each of the four distinct 20 min behavioral observations during the morning and afternoon testing sessions. If behaviors routinely occurred at roughly the same time of day across multiple recordings, this would have suggested that any temporal clumping of these behaviors observed within any particular behavioral observation was due to underlying physiological effects (e.g., brain temperature) [68], resulting from similar circadian patterns rather than contagion.

Next, the temporal patterns of each of the four behaviors were analyzed in the following way, described for yawns: the time between adjacent yawns was calculated (inter-yawn interval) and frequencies of occurrence were binned into 30 s intervals based on the previous work on budgerigars—work which indicated that there would be a higher chance of witnessing contagion when bins were extended to this timeframe [36]. If yawning was contagious, one would expect a bimodal distribution, with a higher frequency of closely spaced yawns (30–60 s) followed by longer intervals, until the occurrence of new priming yawns [69]. Furthermore, following Massen et al. [33], observations were removed from the analysis in cases where a false signal of contagion emerged due to one individual displaying the same behavior multiple times in consecutive bins. This included a total of 20 behavioral observations, spread across yawning (1), stretching (8), scratching (9), and shaking (2).

Next, patterns of contagion were more closely examined by investigating whether behavioral runs occurred with a greater frequency than would be expected by chance. Each 20 min observation was broken into forty 30 s bins, and the frequency of each behavior was determined in a similar fashion to Miller et al. [36] and Massen et al. [33]. To identify non-random distributions across the 30 s intervals, separate runs tests were performed across all observations for each behavior using SPSS for Macintosh (Version 27.0, IBM Corp., Armonk, NY, USA). The generated *Z*-scores are normally distributed, with negative values indicating a greater degree of temporal clustering or clumping (i.e., patterns of both consecutive bins with and without a particular behavior), while positive values indicate a greater than expected level of dispersion. A total of 48 behavioral observations had to be removed due to a behavior being completely absent—spread across yawning (31), stretching (12), scratching (4), and shaking (1).

Lastly, a combined probability test was performed by hand, as described by Sokal and Rohlf [70] (pp. 778–782), to determine the overall probability of non-random clumping or clustering across all of the remaining observations for each behavior, by taking into account the probability values from each of the individual runs tests. Means and standard deviations are reported as descriptive statistics in text, *p*-values were two-tailed, and alpha was set to 0.05 in all analyses.

## 3. Results

### 3.1. Descriptive Analyses and Circadian Effects

**Figure 2:**
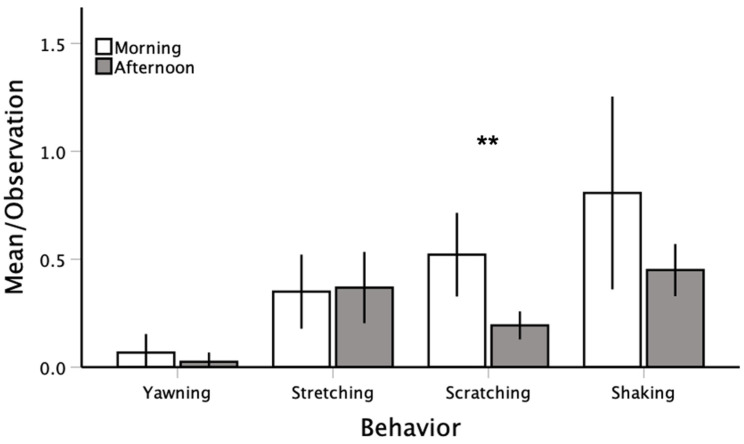
The average behavioral frequency per bird across the morning and afternoon observations. Note: M ± 95% CI; ** *p* < 0.01.

A total of 655 targeted behaviors were observed across the 44 20 min observations. Yawning was by far the most infrequent behavior (*n* = 23), followed by stretching (*n* = 157), scratching (*n* = 177), and shaking (*n* = 298) (Table 2). Per 20 min observation, we noted 0.052 ± 0.076 yawns/bird, 0.357 ± 0.194 stretches/bird, 0.402 ± 0.176 scratches/bird, and 0.677 ± 0.415 shakes/bird. Behaviors tended to occur more frequently in the morning hours compared to the afternoon (see Figure 2), though this was only statistically significant for scratching (yawning: *t* = 1.054, *p* = 0.307; stretching: *t* = −0.210, *p* = 0.838; scratching: *t* = 3.641, *p* = 0.005; shaking: *t* = 1.851, *p* = 0.097). The random effects from the GLMM are reported in Table S1.

### 3.2. Temporal Distribution and Contagion Analyses

**Figure 3:**
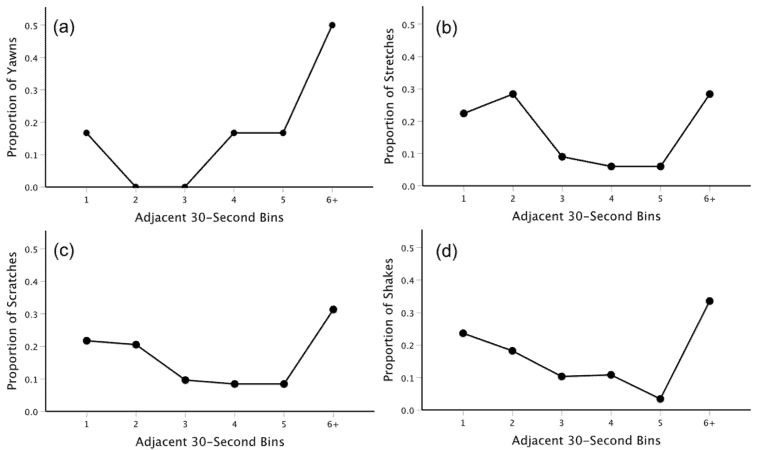
The proportion of yawns (**a**), stretches (**b**), scratches (**c**), and shakes (**d**) that occurred within 30 s bins, ranging from 30- to >180-s, of the most recently performed matched behavior from a separate group member.

**Figure 4:**
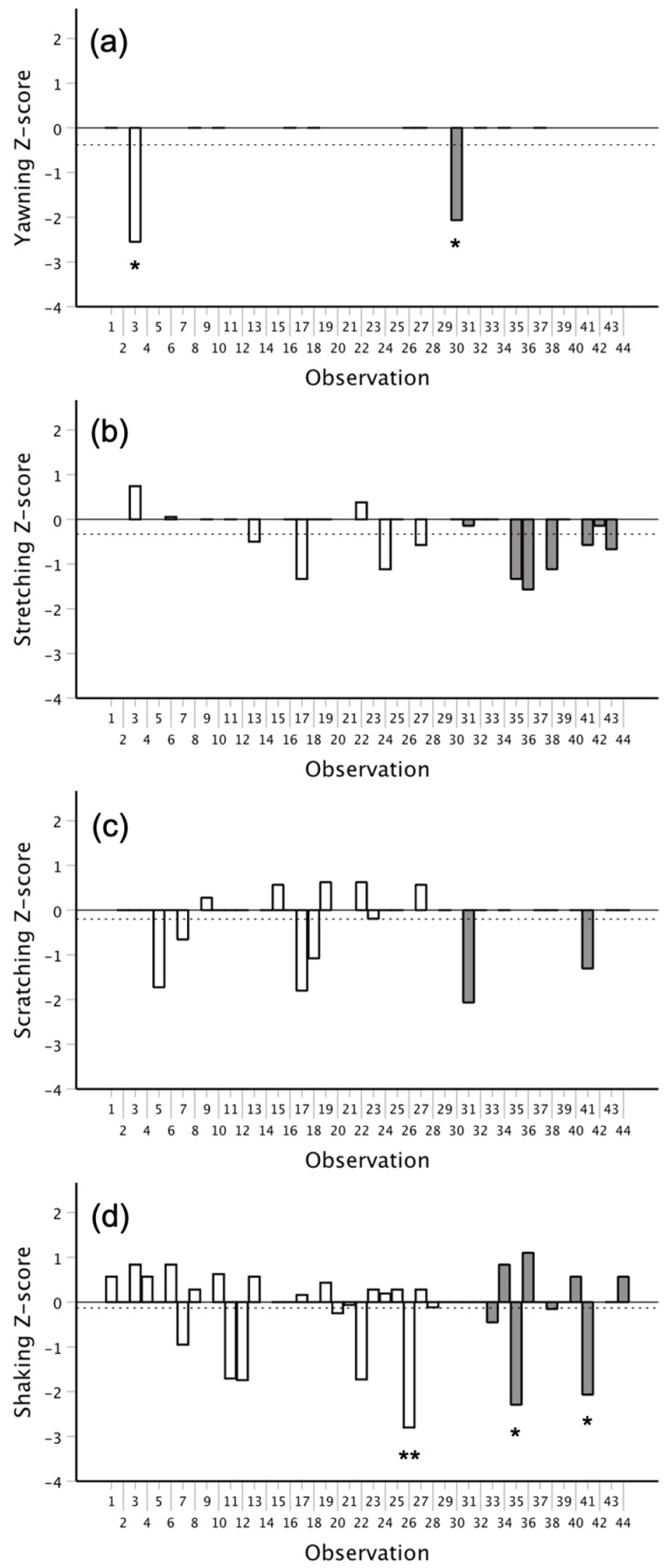
The distribution of *Z*-scores from the runs tests for (**a**) yawning (*n* = 12), (**b**) stretching (*n* = 24), (**c**) scratching (*n* = 31), and (**d**) shaking (*n* = 41) across the morning (white) and afternoon (gray) observations. The dotted line indicates the average *Z*-score across all observations. None of the runs tests revealed significant dispersion, but a few observations showed significant clustering. * *p* < 0.05; ** *p* < 0.01.

Overall, each of the four behaviors was relatively evenly distributed over time across the distinct morning and afternoon observations (see Figure S1). The combined inter-behavioral intervals are plotted in Figure 3. The temporal distributions observed suggested the potential for contagion, as each depicted a U-shaped pattern with a higher frequency of matched behaviors that were both closely spaced in time and separated for longer than 180 s. Figure 4 depicts the runs tests across all 20 min observations for each of the four behaviors. Outputs from these tests revealed that only 2/12 (16.67%) observations for yawning included significant clustering. Moreover, a combined probability test revealed that, across all observations, the degree of clustering among yawns was not significant (*X*^2^(24) = 15.504, *p* = 0.905). Similar effects were observed for each of the other three behaviors. Only 2/24 (8.33%) observations for stretching showed significant clustering, and a combined probability test revealed that the degree of clustering across all observations was not significant (*X*^2^(48) = 28.698, *p* = 0.988). Just 1/31 (3.23%) observations for scratching showed significant clustering, with the combined probability test revealing no significant effect (*X*^2^(62) = 29.483, *p* = 0.999). Lastly, 3/41 (7.32%) observations for shaking showed significant clustering. Again, a combined probability test revealed that, across all observations, the degree of clustering for shaking was not significant (*X*^2^(82) = 61.911, *p* = 0.952).

To test whether the limited clustering we observed was specific to a single time interval (30 s), this analysis was also re-performed when parsing the data into twenty 60 s bins. However, these subsequent results were highly similar, again, showing no evidence for contagion (see Figure S2). Differences in the clustering of behaviors between groups and between morning and afternoon observations were also examined, both for 30 and 60 s bins, but revealed no significant effects (Figures S3 and S4).

## 4. Discussion

This study represents the second attempt to measure contagious yawning and stretching, and the first attempt to measure contagious scratching and shaking, in a species of bird. Despite prior studies reporting various forms of behavioral contagion among ravens [45,46], the current study did not find evidence for contagious yawning, stretching, scratching, or shaking in this species. While each behavior was significantly clustered in time for at least one of the observations, combined probability analyses (taking into account the probability values from across all observations) definitively revealed no overall effect of contagion. This was true when examining both the distribution at 30 s and at the less conservative 60 s bins (see Supplementary Materials).

In addition to addressing behavioral contagion, the current findings also provide the first account of the naturalistic frequency of these behaviors in this species, albeit among small groups in captivity. The observational data collected here suggest that in each hour, ravens yawn 1.6 times, stretch 10.7 times, scratch 12.2 times, and shake 20.3 times, on average. However, there was large individual variability in the expression of these behaviors (Table 2). Yawning and stretching occurred with relatively equal frequency in both the morning and afternoon hours, while scratching and shaking were both more common in the afternoon. In comparison to the budgerigar, in which there is comparable avian data for the relative frequencies of yawning and stretching [36], the rate of stretching for ravens was highly similar, while yawns were only about half as frequent. In fact, the majority of the birds (6/10) in the current study did not yawn a single time across the 44 observations. Additionally, budgerigars displayed an increase in yawn frequency as the day progressed [36], while there was no difference in the frequency of yawning among ravens between the morning and afternoon observations. Whether ravens truly deviate from the pattern observed in budgerigars would require a better investigation of ravens’ activity patterns, particularly since the current study did not encompass many observations in the afternoon, or any between 11:30 and 15:00.

The absence of yawn and stretch contagion in ravens is also in contrast to observational and experimental studies in budgerigars [36,37]. However, potential comparative differences in these responses are to be expected, based on ecological factors and evolutionary history [5]. While ravens are highly gregarious and possess sophisticated social cognition [38,39,52], they live in much smaller groups composed of pair bonds and display less collective behavior in flocking, compared to budgerigars [71]. Given that contagious yawning and stretching are thought to promote motor synchrony [3] and collective vigilance [57,58], this could explain the difference between the two species. Nevertheless, ravens do tend to cooperate in these small parties when scavenging on large prey that is monopolized by pair-bonded individuals or large predators [72], which does require coordination and vigilance that may be enhanced by contagious yawning. Additionally, the social structure of ravens, with their fission–fusion spatial and temporal dynamics [39,73], does resemble that of chimpanzees [74], which do show contagious yawning [8,20,21].

Together, these conflicting comparative findings cast doubt on the purported link between contagious yawning and emotional contagion [8] and suggest that these processes are independent. While some experimental studies have reported emotional contagion among ravens [49,50], to date, there is no evidence for this capacity among budgerigars. Instead, contagious yawning may be tied to bodily synchrony only [75], which budgerigars display when interacting with conspecifics [76].

Given the inherent limitations of observation research, experimental methods should be performed in the future to support the null findings for contagion effects. In particular, the overall occurrence of yawning was quite low, limiting the ability to effectively analyze the social influence of this response. Generally, we cannot rule out that our sample size, though large by the standards of ravens in captivity, was too small to detect a significant effect. To a lesser extent, the same issue could have applied to all the other behaviors as well (though the frequencies for these were much higher). Future experimental research could also examine different time scales at which subsequent behaviors should be considered contagious. Due to the outdoor aviary, there were influential factors to be considered: throughout the observational study, the temperature was not constant, and this is known to influence yawning in birds [77,78]. Parasite load could also have had an influence on scratching levels. Furthermore, the two compartments of the aviary were not completely visually nor acoustically separated; therefore, it is possible that adjacent behaviors between the groups could have occurred and gone unnoticed during the experimental sessions. These factors, however, were not likely to obfuscate our results significantly, since the ravens’ visual access between compartments was still largely obscured.

## 5. Conclusions

Overall, this study represents the second attempt to measure contagious yawning in birds. The temporal analyses presented here do not suggest the presence of contagious yawning, nor any of the other behaviors measured. Given the low frequency of yawning and the limitations of observation research, experimental setups are needed to confirm and clarify these findings, i.e., by using live birds or video recordings as a target stimulus. Nonetheless, this study revealed novel effects with respect to the naturalistic frequency and circadian variation of some everyday behaviors in juvenile common ravens.

## Figures and Tables

**Table 1:** Descriptions of each behavior.

Yawning: a wide opening of the beak combined with a slight closing of the eye.
Stretching: a lifting of one or both wings with extension above the legs and towards the anterior, or a lifting of both wings upwards and backwards.
Scratching: a raising of a leg towards the side and above the wing to brush against the body.
Shaking: a shuddering of the feathers followed by a brief pause in which the feathers are redirected towards their natural positioning.

**Table 2:** Total behavioral frequencies across the individual birds (M = male; F = female).

Individual (Sex)	Yawns	Stretches	Scratches	Shakes
Group 1				
Adele (F)	0	29	26	23
Paul (M)	4	20	34	45
Rufus (M)	8	22	19	74
Max (M)	8	19	8	16
Group 2				
George (M)	0	2	20	15
Horst (M)	0	6	11	17
Laggie (M)	0	18	13	23
Louise (F)	0	8	1	22
Nobel (F)	3	11	13	25
Tom (M)	0	22	19	38
Mean ± SD	2.30 ± 3.335	15.70 ± 7.55	17.70 ± 8.06	29.80 ± 18.26

## Data Availability

The datasets used to generate the results are available here: https://dataverse.harvard.edu/dataset.xhtml?persistentId=doi:10.7910/DVN/BZPTHS (accessed on 25 April 2022).

## Supplementary Materials

The following supporting information can be downloaded at: https://www.mdpi.com/article/10.3390/ani12111357/s1, Table S1: Random effects from the Generalized Linear Mixed Model; Figure S1: The temporal distribution of (a,b) yawning, (c,d) stretching, (e,f) scratching, and (g,h) shaking across the two 20-min morning and two 20-min afternoon observation periods, depicted within 5-min intervals; Figure S2: The distribution of *Z*-scores from the 60-s runs test analyses for (a) yawning, (b) stretching, (c) scratching, and (d) shaking across all morning (white) and afternoon (black) observations; Figure S3: The mean *Z*-scores from the 30- and 60-s runs test analyses between Group 1 and Group 2; Figure S4: The mean *Z*-scores from the 30- and 60-s runs test analyses between morning and afternoon observations.
