# Supplementary material for: No Evidence for Contagious Yawning in Juvenile Ravens (Corvus corax): An Observational Study
Source: Animals (Basel). 2022 May 26;12(11):1357. doi: 10.3390/ani12111357 (PMC9179381; doi:10.3390/ani12111357)
Supplement: Supplementary file 1 [file animals-12-01357-s001.zip › animals-1720799-supplementary.pdf]

Table S1. Random effects from the Generalized Linear Mixed Model

Yawning:

Groups	Name	Variance	Std.Dev.
BIRD:GROUP	(Intercept)	0.000000	0.00000
GROUP	(Intercept)	0.002006	0.04479

Stretching:

Groups	Name	Variance	Std.Dev.
BIRD:GROUP	(Intercept)	0.009198	0.09591
GROUP	(Intercept)	0.017949	0.13397

Scratch:

Groups	Name	Variance	Std.Dev.
BIRD:GROUP	(Intercept)	3.526e-03	5.938e-02
GROUP	(Intercept)	2.354e-10	1.534e-05

Shake:

Groups	Name	Variance	Std.Dev.
BIRD:GROUP	(Intercept)	0.01919	0.1385
GROUP	(Intercept)	0.01825	0.1351

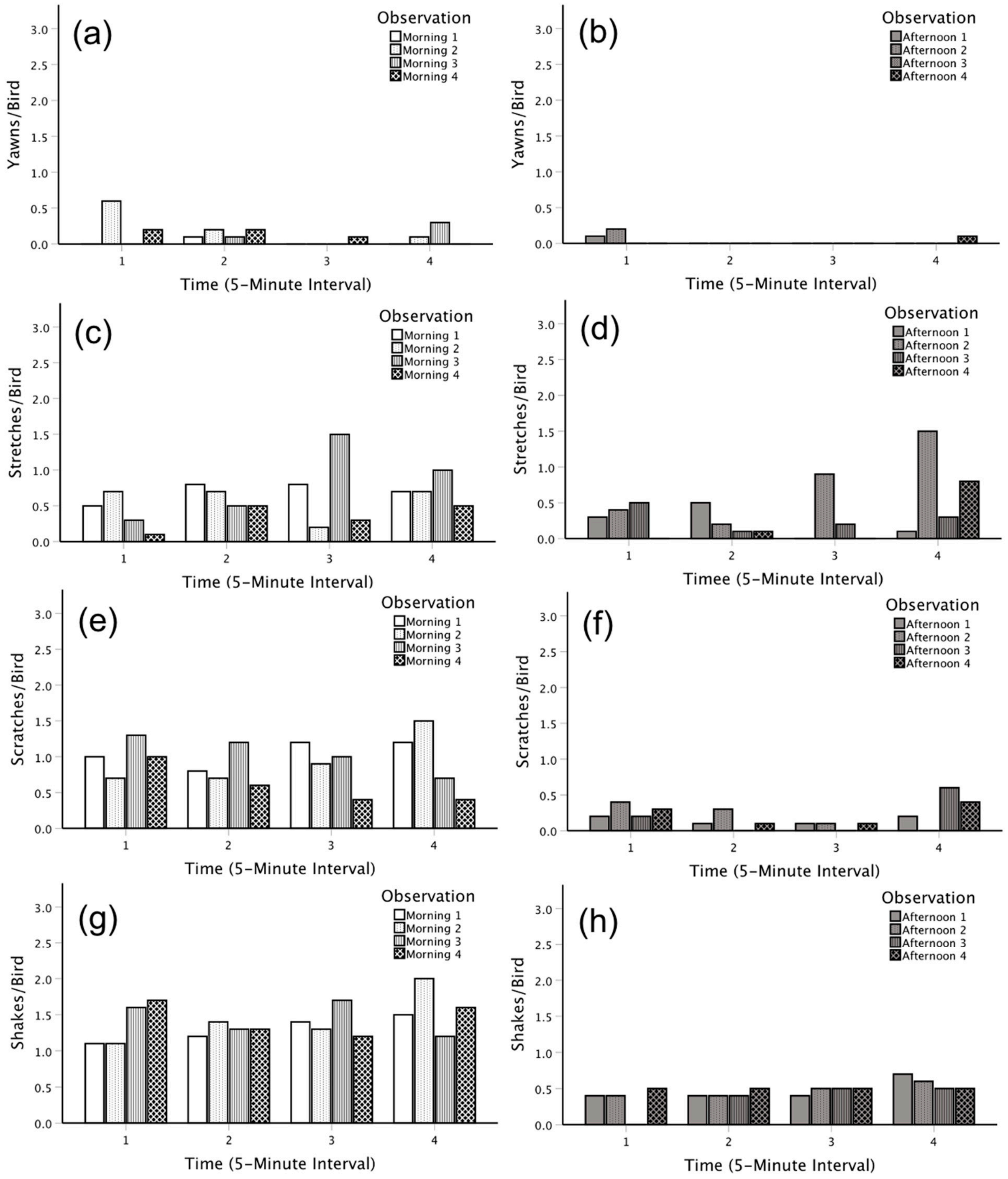

Figure S1. The temporal distribution of (**a,b**) yawning, (**c,d**) stretching, (**e,f**) scratching, and (**g,h**) shaking across the two 20-minute morning and two 20-minute afternoon observation periods depicted within 5-minute intervals.

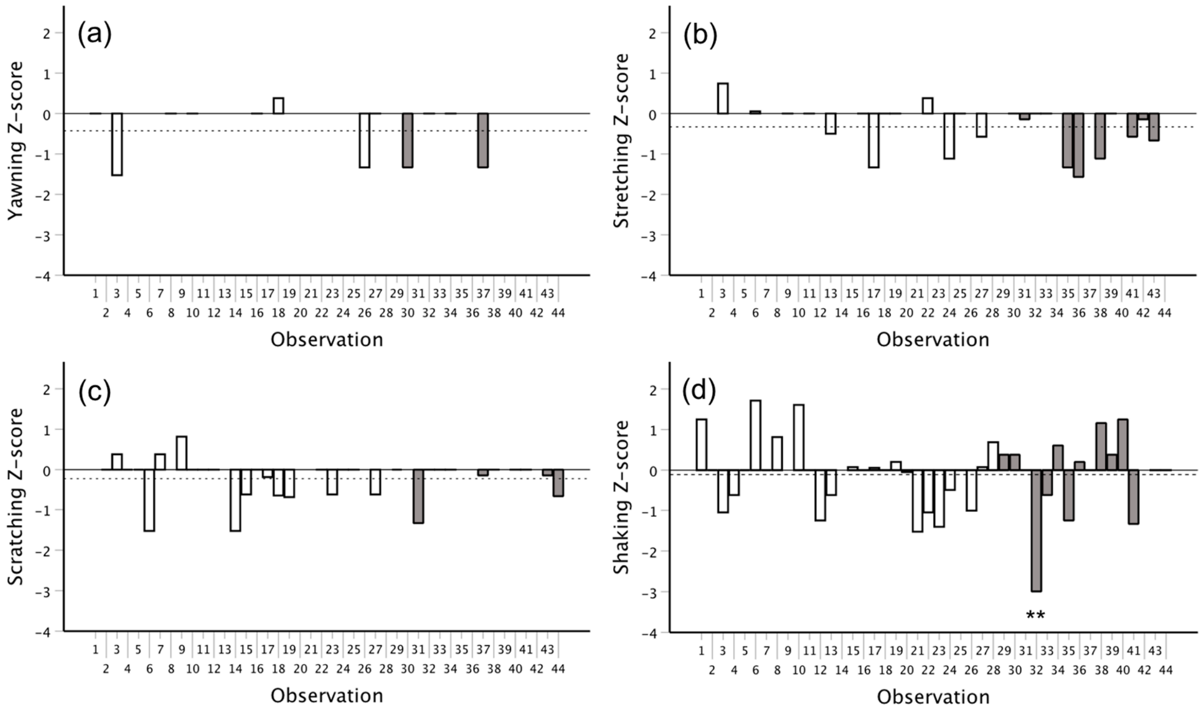

Figure S2. The distribution of *Z*-scores from the 60-second runs test analyses for (**a**) yawning, (**b**) stretching, (**c**) scratching, and (**d**) shaking across all morning (white) and afternoon (gray) observations. For yawning, 0/12 runs tests were significant; combined probability test ($X^2(24) = 15.053$, $p = 0.919$). For stretching, 0/24 runs tests were significant; combined probability test ($X^2(48) = 23.583$, $p = 0.998$). For scratching, 0/31 runs tests were significant; combined probability test ($X^2(62) = 23.526$, $p = 0.999$). For shaking, 1/41 runs tests were significant; combined probability test ($X^2(82) = 66.967$, $p = 0.885$).

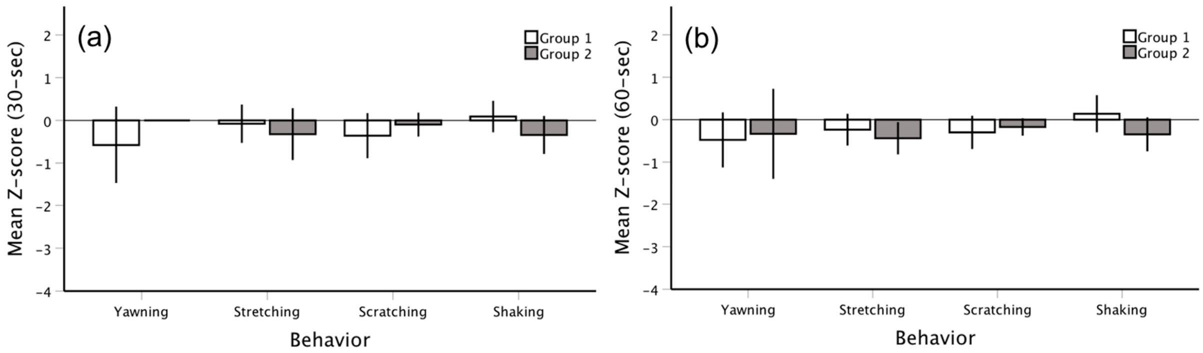

Figure S3. The mean *Z*-scores from the 30- (**a**) and 60-second (**b**) runs test analyses between Group 1 and Group 2. The level of temporal clustering did not vary as a function of group compositions. Note: $M \pm 95\%$ CI; all p-values > 0.05.

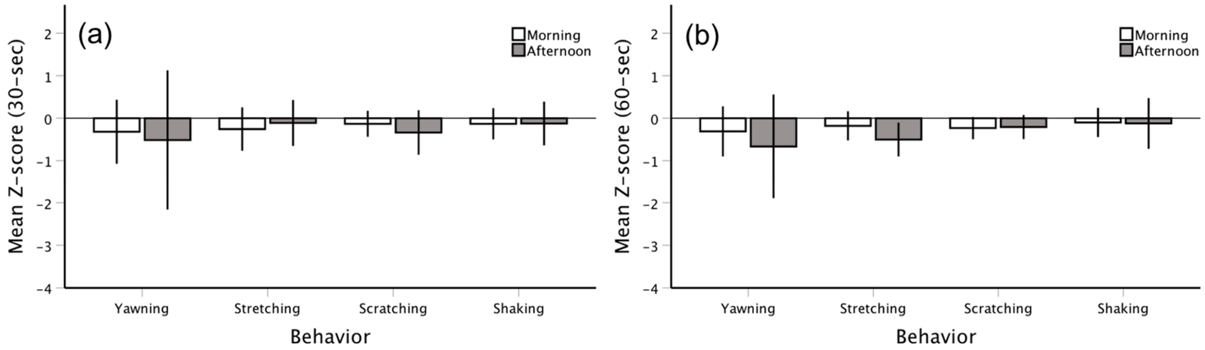

Figure S4. The mean *Z*-scores from the 30- (**a**) and 60-second (**b**) runs test analyses between morning and afternoon observations. The level of temporal clustering did not vary as a function of the time of the observations. Note: M ± 95% CI; all p-values > 0.05.
